# Special Issue “Strategies to Develop High-Quality Gluten-Free Products Welcomed by Consumers”

**DOI:** 10.3390/foods12142803

**Published:** 2023-07-24

**Authors:** Hiroyuki Yano

**Affiliations:** Institute of Food Research, National Agriculture and Food Research Organization, Tsukuba 305-8642, Japan; hyano@affrc.go.jp

Extensive and long-term efforts on wheat breeding [1] have made wheat flour a useful material in making bread, noodles, and other various foods worldwide [2]. Nowadays, wheat is regarded as one of the representative food ingredients, and the great versatility of wheat gluten has made it difficult for consumers to find food devoid of any gluten. However, due to the prevalence of celiac disease/wheat allergy, as well as the changing dietary lifestyle, the demand for gluten-free foods is expanding [3]. This Special Issue called for papers written in view of the strategies to develop high-quality gluten-free food products that meet the demands of consumers. Herein, five articles/communication papers and three reviews/perspectives cover a broad spectrum of information, categorized into two major key strategies (Figure 1):

A. The development of hypoallergenic wheat lines, which possess equivalent food processing abilities to that of conventional wheat lines. 

B. The replacement of wheat with rice, soybean, or other gluten-free ingredients.

## 1. Strategy A

Dr. Morita’s group has thus far developed a non-transgenic, hypoallergenic line from wheat cultivar Hokushin [12]. The Hokushin 1BS-18 (1BS-18H) lacks ω5-gliadin, a major allergen responsible for wheat-dependent exercise-induced anaphylaxis (WDEIA). The intravenous challenge of the ω5-gliadin-deficient 1BS-18H gluten did not elicit allergenic reactions in a wheat allergy rat model [13]. Moreover, a dough made from the flour of 1BS-18H wheat retained equivalent viscoelastic properties to a dough made from conventional wheat flour [14]. In this Special Issue, Yamada et al. [4] report that 1BS-18H has induced oral tolerance to wheat gluten proteins in a rat model of wheat allergy. The series of studies have shed new light on the prevention of wheat allergy.

Recently, a variety of approaches have been undertaken to develop hypoallergenic wheat products edible for patients with IgE-mediated wheat allergies. In a narrative review, Morita et al. [5] summarized the current status of these studies and introduced examples of wheat products, which significantly reduced the reactivity of serum IgE in wheat allergic patients. Hypoallergenic wheat could also be used for the development of immunotherapy protocols for desensitizing patients to specific wheat allergens. On the other hand, the review also addresses challenges faced in creating hypoallergenic wheat lines for wheat-allergic patients by either traditional breeding or biotechnology approaches.

## 2. Strategy B

Other approaches include producing gluten-free food products which have been made using wheat flour as a major ingredient. Yano and Fu [6] reviewed recent studies on the development of gluten-free foods in which plant proteins are utilized effectively. The review also introduces the particle-foam mechanism, which realizes the additive-free, gluten-free rice bread. Furthermore, in the case of the egg-white/soy protein bread, disulfide exchanges play critical roles on the swelling mechanism. Fujii et al. [7] provided a valuable clue on the long-standing problem of rice bread: a short shelf life due to its hardening over time. They found that the addition of modified tamarind gum/tamarind gum or xanthan gum effectively alleviated the hardening of rice bread. Moreover, modified tamarind gum was effective in improving the quality of rice bread, such as an increased softness and preservation of moisture. In the development of gluten-free rice noodles, Sugiyama et al. [8] focused on the taste buds of Japanese consumers. First, they made Koshihikari white rice flour and potato starch into noodles with a chewy texture similar to that of Udon noodles. Then, the authors developed a roasted brown rice version, which has a taste suitable for the Japanese population. The study is interesting in that it pursued the texture and flavor profile preferred by a specific population. In addition, the step-by-step approaches were supported by scientific analyses, including mechanical tests (stress–strain characteristics) and GC-MS analyses (aroma characteristics). Other than rice, Maeta et al. [9] provided new supportive evidence for soybean as a suitable wheat-replacing food ingredient. From a nutritional point of view, soybean powder had a 2- and 12-folds higher polyphenol content and oxygen radical absorbance capacity (ORAC), respectively, than bread flour. Furthermore, using 44 healthy human subjects, satiety maintenance and hunger suppression capacity were compared after the ingestion of soybean or wheat bread powder. It was found that soybean had a higher relative “effect size” for maintaining satiety (+0.274) as well as a suppression of hunger (−0.341)/appetite (−0.424) compared to wheat bread powder. The authors concluded that soybean is expected to be a useful gluten-free ingredient in terms of both nutritive value and physical effect. Koriyama et al. [10] report that roasting soybeans at 190 °C or higher increases the 1,1-diphenyl-2-pucrylhydrazy (DPPH) radical scavenging activity as well as oxygen radical absorbance capacity value. Before roasting, soybeans with the darkest seed color had the strongest antioxidant activity. However, the degree of increased antioxidant activity by roasting was almost similar regardless of the seed coat color. Moreover, in the case of aged beans, while DPPH radical scavenging activity increased in yellow soybeans and decreased in green/black soybeans when in storage for 2 months, roasting significantly increased the radical scavenging activity of all beans. The authors recommend roasting soybeans for an improved antioxidant activity, particularly in the case of aged beans. A review on hemp studies summarizes its sustainable growth characteristics, high nutritive value of the seeds, and unique protein feature in food processing [11]. The cultivation of hemp is low-cost and environmentally friendly. The high SH (sulfhydryl)/S-S (disulfide) rate of hemp protein facilitates sulfhydryl-disulfide exchange reactions with high S-S/SH protein counterparts from other sources such as soybean. The “donor-recipient theory” appears to be one of the key mechanisms of food processing in the development of new foods.

## 3. Conclusions

**Figure 2:**
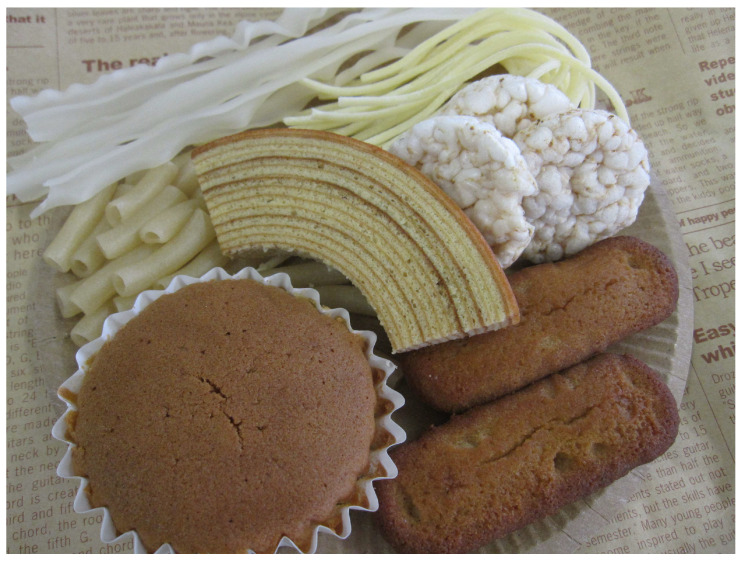
Examples of gluten-free food products available on the market.

At present, copious scientific journals, including *Foods,* have various Special Issues featuring gluten-free products, as well as the pros and cons of a gluten-free lifestyle. Research on the development of gluten-free foods will surely expand in the future. While wheat products currently provide 20% of calories and protein for people worldwide [2], it is without a doubt that the ongoing diversification of food production will gradually result in the replacement of wheat with rice, soybeans, hemp, and others. While several gluten-free products are already on the market (Figure 2), they remain to be within an evolutionary stage. The effective use of sustainable plants with a reduced cultivation cost/ecological burden, as well as waste products such as oil cakes, will proceed to abide by the Sustainable Development Goals. Besides, an increasing risk of disasters urges researchers to produce long-life and portable (lightweight) gluten-free foods with high nutritive value to be easily distributed within safe shelters. The Editor closes this Special Issue with great anticipation for the further development of high-quality gluten-free foods which are able to win the hearts of all consumers.

## Figures and Tables

**Figure 1:**
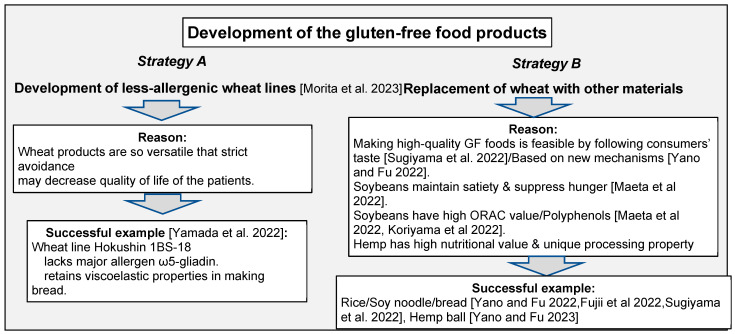
Strategies in developing gluten-free products addressed in this Special Issue [4,5,6,7,8,9,10,11].
